# Electron Diffraction (Micro-ED): A dedicated device and its applications in the pharmaceutical industry

**DOI:** 10.1063/4.0000913

**Published:** 2025-10-27

**Authors:** Gustavo Santiso-Q, Christian Jandl, Johannes Merkelbach, Laura Samperisi, Gunther Steinfeld s, Danny Stam

**Affiliations:** Eldico Scientific, Allschwil, Basel-Landschaft, Switzerland

## Abstract

Since the Science nomination for “Breakthrough of the year 2018” [1], electron diffraction (3D ED, known also as micro-ED) for structural elucidation of organic compounds has been gaining a lot of momentum. 3D ED has been rapidly evolving as a complementary technique for SCXRD experiments [2]. Especially for those cases where single crystals of suitable size for XRD experiments are not obtained. Dedicated electron diffractometers [3] make the technique more accessible, resulting in many recent publications where these commercial electron diffractometers have been used [4].

3D ED / Micro-ED’s ease of use and its potential for various applications is presented in multiple studies, especially in the pharmaceutical industry where it is having a high impact [5,6]. We would highlight some case studies where electron diffraction has helped the pharmaceutical industry obtain structural information not available before by other means [4b, 6]. Furthermore, we’ll delve and showcase other applications like: Characterization of meta-stable polymorphic forms, identifying crystallinity in amorphous solid dispersions, finding new polymorphic forms, identifying polymorphic forms for litigation purposes, impurities and phase identification using crystal mapping, and more.
